# Pharmacokinetics, tissue distribution, and antitumor activity of a novel compound, NY-2, in non-small cell lung cancer

**DOI:** 10.3389/fphar.2022.1074576

**Published:** 2023-01-16

**Authors:** Yingshi Zhang, Chang Xu, Xiangbo Xu, Lingxiang Ma, Ruolan Li, Zihua Xu, Qingchun Zhao

**Affiliations:** ^1^ Department of Pharmacy, General Hospital of Northern Theater Command, Shenyang, Liaoning, China; ^2^ Department of Clinical Pharmacy, Shenyang Pharmaceutical University, Shenyang, Liaoning, China; ^3^ Company of Beigene Biotechnology Co., Ltd., Beijing, China

**Keywords:** NY-2, pharmacokinetics, tissue distribution, antitumor activity, non-small cell lung cancer

## Abstract

**Introduction:** ZLDI-8, which has a relatively strong antitumor activity, is an inhibitor of ADAM-17 and acts on the Notch signaling pathway. To further optimize its structure and improve its activity, a series of derivatives of ZLDI-8 was synthesized. NY-2 was the most effective derivative based on preliminary activity screening in vitro, with no obvious toxicity after administration *in vivo*.

**Method:** The study aimed to determine the pharmacokinetics, tissue distribution, hepatotoxicity, nephrotoxicity, and antitumor activity of compound NY-2 on non-small cell lung cancer (NSCLC) *in vitro* and *in vivo*.

**Results:** The in vivo pharmacokinetics parameters of NY-2 were better than those of ZLDI-8. The tissue distribution analysis showed that tail vein injection of 6 mg/kg of NY-2 in rats resulted in the highest concentration in the lung, so we hypothesized that NY-2 might be effective in the treatment of non-small cell lung cancer. In vitro assays showed that NY-2 significantly inhibited tumor colony formation, invasion, and migration and increased LDH activity and apoptosis in a concentration-dependent manner in non-small cell lung cancer cells. NY-2 also inhibited the formation of lung metastases without significant toxicity to major organs in nude mice.

**Conclusion:** Compared with the parent compound, ZLDI-8, the activity and safety of NY-2 were higher. NY-2 acts on ADAM17 and simultaneously affects the downstream Notch1 and integrinβ1 signaling pathways resulting in antitumor activity. Thus, NY-2 could be a potential antitumor agent, inhibiting the organization and development of non-small cell lung cancer.

## Introduction

The occurrence, development, and metastasis of cancer is still a huge burden worldwide with increasing morbidity and mortality. Lung cancer has the highest mortality rate among all malignancies, causing more than 350 deaths every day with a 5-year survival rate of only 22%, far lower than other malignancies, such as breast, colorectal, and cervical cancer (Siegel et al., 2022; Xia et al., 2022). Lung cancer can be divided into non-small cell lung cancer (NSCLC) and small cell lung cancer (SCLC) categories according to histopathological features. NSCLC accounts for 80%–85% of all lung cancers, and NSCLC can be subdivided into adenocarcinoma, squamous cell carcinoma, and large cell carcinoma (Xie et al., 2021). Due to the lack of specific symptoms and detection indicators in the early stages of NSCLC, most patients are already in the middle and advanced stages when they are clinically diagnosed (Vinas et al., 2016). The overall survival of NSCLC patients has significantly increased in recent years under the combined effect of thoracoscopic surgery, targeted therapy, and immunotherapy and the improvement in overall survival has also been closely related to the increased use of screening (Chang et al., 2021; Liang et al., 2021; Duan et al., 2022). At present, platinum-based chemotherapy is still the standard treatment regimen for patients with advanced NSCLC. However, the overall efficacy is relatively low and the adverse reactions such as nausea, vomiting, and fatigue are serious. Therefore, it is of great importance to find agents for new treatment targets, with high efficiency and low toxicity for the clinical treatment of NSCLC (Liang et al., 2020; Jiang et al., 2021; Katakura and Murakami, 2022; Liu et al., 2022; Pang et al., 2022).

**FIGURE 1:**
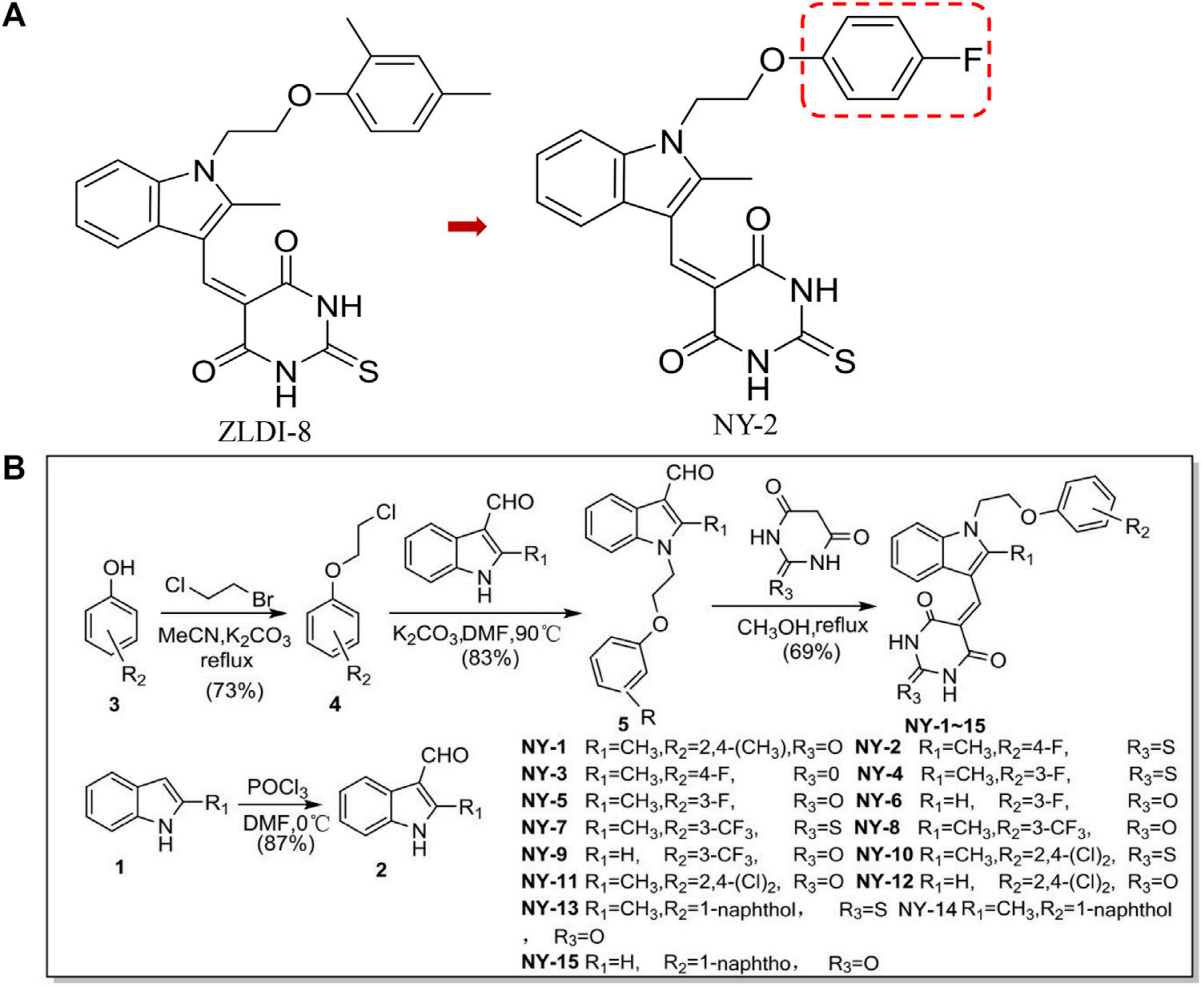
Chemical structures of ZLDI-8 and NY-2 **(A)**. Chemical structures of the derivatives, NY-1 to -15 **(B)**.

Excessive activation of the Notch signaling pathway may be involved in the induction, development, and metastasis of NSCLC (Augert et al., 2019; Sharif et al., 2020; Wang et al., 2020; Zhang et al., 2021). Notch signaling is activated when ADAM17 (a disintegrin and metalloprotease17) cleaves off its extracellular segment in the first step and γ-secretase in the second step (Zhou et al., 2022). Activated Notch promotes the epithelial-mesenchymal transition (EMT) of tumor cells, resulting in distant metastases or recurrence of NSCLC and treatment failure (Kar et al., 2019; Wang et al., 2019; Feng et al., 2021). In preliminary work from our research group, molecular docking experiments were carried out for ADAM17, and an active compound, ZLDI-8, was found. ZLDI-8 had high antitumor activity against many malignancies such as colorectal cancer, hepatocellular carcinoma, and NSCLC, and could also inhibit activation of the Notch signaling pathway and block the EMT (Li et al., 2018; Zhang et al., 2018; Lu et al., 2019). However, there are still many aspects to be further studied. The pharmacological properties of ZLDI-8, the pharmacokinetics of absorption, distribution, and excretion in vivo, and tissue distribution have not been systematically evaluated. To optimize and improve its activity, we modified the structure of the hydrophobic region of the 2-iliacbibcibic acid. Halogen atoms, due to their strong electronegativity, can enhance the binding of a compound to its targets and improve pharmacological activity (Wilcken et al., 2013; Amato et al., 2014). Therefore, we attempted to optimize the derivative by the addition of halogen atoms or halogen-containing groups into the hydrophobic region of ZLDI-8 (Li et al., 2015; Mardirossian et al., 2021) and designed and synthesized 15 derivatives, NY-1 to 15 (Ning, 2018), with the structure shown in Figure 1.

We tested the antitumor activity of the 15 ZLDI-8 derivatives, and found a lead compound, NY-2, with strong antitumor activity. NY-2 was then evaluated by a comparative in vivo study in rats of hepatotoxicity and the pharmacokinetics and tissue distribution of the compound. This provided solid material evidence and laid a theoretical foundation for ZLDI-8 and its derivative NY-2 as antitumor agents. The tissue distribution analysis showed that, after tail vein administration, the concentration of NY-2 was highest in the lung. Thus we hypothesized that NY-2 might be effective as a chemotherapeutic agent in treating lung cancer. The in vivo and in vitro activities of NY-2 against NSCLC were evaluated in terms of cell viability, apoptosis, invasion, and migration, and its effect on the tumorigenesis of lung metastases in a nude mouse model.

## Methods

### Cell culture and reagents

The human colorectal cancer cell lines, HCT116 and LoVo, the breast cancer cell line, T47D, the hepatocellular carcinoma cell line, HepG2, the normal human liver cell line, HL7702, human umbilical vein endothelial cells (HUVECs), and the non-small cell lung cancer cell lines, A549, H1993, HCC827, H460, were purchased from the American Type Culture Collection (Manassas, VA, United States). Cell lines were cultured in the recommended RPMI-1640 medium (AE24464298, Hyclone, United States) and DMEM medium (AE29422278, Hyclone, United States) supplemented with 10% fetal bovine serum (FBS; JC63470, FB15015, Clark, United States) with 1% penicillin-streptomycin. Cells were maintained in an incubator at 37°C with 5% CO_2_ (HF90,Sanyo, Japan) and passaged every 48–72 h, after detaching with 0.25% trypsin (Sigma, United States). The compounds ZLDI-8 and NY-1 to -15 were synthesized in the laboratory of Dr. Huaiwei Ding in Shenyang Pharmaceutical University, and verified by high-performance liquid chromatography (purity >95%) for use in experiments (Supplementary Figure S1). Paclitaxel was purchased from MedChemExpress (HY-B0015, 99.97% purity, United States).

### Animals, hepatotoxicity, and nephrotoxicity experiments

Animal experiments were approved by the Ethics Committee on Laboratory Animal Management of Shenyang Pharmaceutical University (Approval Document No. SYPU-SQ-001). All animals received humane care according to the local guidelines for the Care and Use of Laboratory Animals of the Shenyang Pharmaceutical University. All animals were housed under specific pathogen-free (SPF) conditions at 25°C with 50% humidity, with free access to food and water for 7 days to adapt. KM mice (male, 4–5 weeks old and weighing 18–22 g) were purchased from Liaoning Changsheng Biotechnology Co., Ltd. (Liaoning, China, animal license #SCXK-2020–0001). For the hepatotoxicity and nephrotoxicity assays, NY-2 was injected via tail vein into KM mice, and the results were used in deciding on the test dose for antitumor activity in a nude mouse cancer model. Its influence on the immune system was also determined. The treatment groups were ZLDI-8, NY-2, DMSO control, and normal saline control, with six mice in each group. The mice were fasted for 12 h before the experiment, but were allowed to drink water freely. ZLDI-8 and NY-2 were dissolved in 1% DMSO containing 1% Tween and 6 mg/kg was administered by a daily tail vein injection for seven consecutive days (Lu et al., 2020).

After injection of the compounds, blood was drawn and serum was collected for the determination of ALT and AST levels (Nanjing Jiancheng Bioengineering Institute, China, C009-2-1 and C010-2-1) to assess liver function. Serum creatinine (sCr, Nanjing Jiancheng Bioengineering Institute, China, C011-2-1) and blood urea nitrogen (BUN, Nanjing Jiancheng Bioengineering Institute, China, C013-2-1) were measured to assess renal function. Tumor necrosis factor-α (TNF-α, Cusabio, Wuhan, China, CSB-E04741m) and interleukin-6 (IL-6, Cusabio, Wuhan, China, CSB-E04639m) were used as markers to evaluate the immune inflammatory response, and an assay kit (T-AOC, S0119, Beyotime) was used to measure total antioxidant capacity by the ABTS method. Mice were euthanized by cervical dislocation, liver and kidney tissue was removed and fixed in formalin, dehydrated in ethanol, embedded in paraffin, and sectioned for staining with hematoxylin and eosin (H&E).

### Pharmacokinetics

We used the online ADME prediction tool Swiss AMDE (http://www.swissadme.ch/index.php) (SwissADME, 2017) to analyze the physicochemical descriptors and ADME parameters of NY-2 and ZLDI-8, to predict their pharmacokinetic properties, drug-like nature, and medicinal chemistry. Compared with ZLDI-8, the log PO/W of NY-2 was increased slightly after introducing fluorine in the para position of the benzene ring, but other properties were basically the same. It is worth noting that while ZLDI-8 is a P-gp substrate, NY-2 is not, which is beneficial for increasing its therapeutic effect on drug-resistant cancer cells. Male Sprague-Dawley rats weighing 240 ± 20 g were purchased from Liaoning Changsheng Biotechnology Co., Ltd. (Liaoning, China, animal license #SCXK-2015-0001). Rats were used for the tissue distribution and pharmacokinetics experiments because of their larger body fluid volume. The rats were fasted for 12 h before the experiment but allowed to drink water freely. Animals were anesthetized with 4% chloral hydrate (0.5 mL/100 g), the total blood volume was collected from the abdominal aorta and quickly dispensed into heparinized EP tubes prepared in advance. The rats were euthanized after blood collection was completed. Plasma samples were subjected to a protein precipitation method for the quantitative analysis of ZLDI-8 and NY-2 by LC-MS/MS (API 3200MD, AB Sciex, United States). The samples were injected into the ionization source by the auto-sampler. A series of standard solutions of ZLDI-8 were prepared with final concentrations of 20, 50, 100, 250, 500, 1000, and 1500 ng/mL; and NY-2 was prepared with final concentrations of 100, 200, 500, 800, 1200, 1600, and 2000 ng/mL. The linear equation and correlation coefficient (r) of the regression curve were calculated from these concentrations and the corresponding absorption data. The specificity, lower limit of quantification (LLOQ), linear range, precision, accuracy, matrix effects, extraction recovery, stability of analytes during the whole process of storage, as well as treatment in a biological matrix and solution were determined to verify the reliability of the compound concentrations *in vivo*.

### Tissue distribution

After fasting for 12 h, 32 SD rats were randomly divided into eight groups of four rats each. Four groups were injected with ZLDI-8 via tail vein (6 mg/kg) at 5 min, 12, 24, and 48 h. The other four groups were injected with NY-2 (6 mg/kg) at the same time points. The heart, liver, spleen, lung, kidney, brain, muscle, and intestines were collected after cervical dislocation at the specified time points and tissue samples were removed. Samples were rinsed with normal saline at 0°C to remove residual blood and debris, blotted dry with filter paper, weighed, and stored at ˗20°C until used.

### Cell viability, lactate dehydrogenase (LDH), cell migration, and invasion assays

Cell viability was measured by the MTT assay. HCT116, HL7702, A549, H1993, HCC827, and H460 cells were dispensed into 96-well plates at a density of 1 × 10^5^ cells/mL. After 24–48 h incubation, cells were treated with the compounds at different concentrations for the indicated periods. An assay kit (WLA073, Wanlei Co., Liaoning, China), was used according to the manufacturer’s instructions to measure LDH activity, The absorbance at 450 nm was measured with a microplate reader (Elx-800, Bio-Rad, China).

For the transwell invasion assay, Matrigel (50 mg/L) was diluted 1:8 with serum-free medium, and 60 μL of diluted Matrigel was pipetted into each transwell (Corning, NY, United States) and incubated overnight. The upper chamber was seeded with 1× 10^5^ cells that had been starved for 24 h in 100 µL of serum-free medium, and 700 µL of medium with 10% FBS was added to the lower compartment. After cultivation for 48 h at 37°C, the cells in the upper chamber were removed with a cotton swab, and the cells on the lower surface of the filter were fixed with methanol for 20 min and stained with 0.1% crystal violet after drying. Excess stain was eluted with 33% acetic acid, and the absorbance was measured at 490 nm.

A wound-healing assay was used to assess the migration ability. Cells were seeded into 6-well plates at 3 × 10^5^ cells per well. At about 90% confluence, uniform scratches were made in the monolayer using a sterile 200 μL pipet tip. After washing with phosphate-buffered saline (PBS), the cells were cultured in 2% FBS medium and photos were taken at 0 and 48 h. The rate of cell migration was determined by measuring the decrease in gap with compared to the initial gap at 0 h. Readings were performed independently in triplicate.

### Colony formation, apoptosis, and western blot protein analysis

For the colony formation assay, cells were seeded into 6-well plates at 500 cells per well, and allowed to attach for 4–6 h at 37°C. The cells were incubated with different concentrations (2.5, 5, and 10 μM) of NY-2 for 6 days with culture medium changed every 48 h. The colonies were stained with 0.1% crystal violet, decolorized with 33% glacial acetic acid, and counted.

For the apoptosis assay, cells were seeded into 6-well plates at 1× 10^5^ cells per well, and after attachment for 4–6 h, they were incubated with different concentration of NY-2 for 48 h. After washing with 1×PBS, the cells were stained with Hoechst 33342 (5 μg/mL, 1 mL per well, C1022, Beyotime Biotechnology, China) for 20 min in a 37°C incubator followed by two washes with PBS. The number of apoptotic cells was counted under an inverted fluorescence microscope (Olympus, Japan). Apoptosis was also determined using an annexin V-FITC apoptosis detection kit (ABS50001, Absin Bioscience Inc., Shanghai, China). Cells were plated in 100 mm Petri dishes at a density of 1 × 10^6^ cells/dish, after 4–6 h for attachment, cells were incubated with different concentrations of NY-2 for 48 h. For each sample, cells were harvested by trypsin digestion without EDTA and washed with 1 × PBS. Cells were resuspended in 300 μL of binding buffer, and 5 μL of annexin V-FITC solution were added and incubated at room temperature in the dark for 15 min, followed by the addition of 5 μL of propidium iodide (PI) solution. The sample staining was assessed by flow cytometry (BD Co., United States).

Antibodies against ADAM17 (Cat. No. ab39163), Notch1 (Cat. No. ab52627), integrin β1 (Cat. No. ab52971), β-actin (Cat. No. ab8226), and anti-rabbit IgG (Cat. No. ab6728) and anti-mouse IgG (Cat. No. ab190475) secondary antibodies conjugated with horseradish peroxidase (HRP) were purchased from Abcam (Cambridge, United Kingdom). Total protein was extracted from HCC cells, and samples were separated by SDS-PAGE and blotted to polyvinylidene fluoride (PVDF) membranes (Millipore, Billerica, MA, United States). The membranes were blocked and then incubated with primary antibodies, followed by washing and incubation with HRP-conjugated secondary antibodies. Stained blots were developed with enhanced chemiluminescence reagents (Pierce, Rockford, IL, United States) and imaged on X-ray film.

### Xenograft NSCLC metastasis model

Healthy BALB/c nude mice (male, 4–5 weeks old, weighing 18–22 g) were purchased from Beijing Huafu Biotechnology Co., Ltd. (Beijing, China, animal license #: SCXK-2019-0008). The experimental groups included the normal (no tumor cells, vehicle) group, the model (tumor cells, vehicle) group, NY-2-treated, and paclitaxel-treated groups, with six mice in each group. After 7 days for adaptation, 200 µL of A549 cells (2× 10^6^ cells/mice) in serum-free medium were injected into the tail vein except for those in the normal group. Drug or vehicle administration *via* tail vein was started on the next day. The normal group and model group were given the same amount of sterile water containing 1% DMSO and 1% Tween as vehicle control. NY-2 was administered at a dose of 6 mg/kg, paclitaxel was administered at a dose of 1 mg/kg (Lu et al., 2020). Injections were done every other day. The general conditions of the nude mice, with regard to activity and food ingestion, were observed every day, and their body weight was measured every other day. After 28 days, the nude mice were euthanized by cervical dislocation, and the tumor nodules on the surface of the left lung were counted after staining with Bouin’s solution (Lu et al., 2020).The right lung, heart, liver, and kidney were removed and fixed in 4% paraformaldehyde then sectioned and stained with H&E.

### H&E tissue staining

KM mice were treated with ZLDI-8 or NY-2 by tail vein injection (6 mg/kg) for 7 days, then the mice were euthanized and liver and kidney tissues were removed and sectioned. For the experiment with BALB/c nude mice, the tail vein injections of NY-2 (6 mg/kg) were continued for 28 days, and the lungs, tumor, heart, liver, and kidney were removed. Trimmed tissue samples were fixed in 4% paraformaldehyde for 24 h, dehydrated in graded ethanol solutions, embedded in paraffin and sectioned. Sections for staining were dewaxed with xylene and then rehydrated, and hematoxylin-eosin staining was performed, after which the sections were again dehydrated in ethanol and cleared with xylene before being mounted on a slide, cover-slipped and examined microscopically. The remainder of the tumors and tissues was frozen in liquid nitrogen for other studies.

### Statistical analysis

The plasma concentration-time concentration data of ZLDI-8 and NY-2 at different times were fitted by DAS2.0 pharmacokinetics software and the drug-time curve was drawn. The drug-time curves of the two compounds were compared and simulated, and a two-compartment model was selected to calculate the pharmacokinetic parameters using the statistical moment algorithm. The peak value (C_max_), half-life (t_1/2_), area under the curve (AUC_0-t_ and AUC_0-∞_), plasma clearance rate (CL), mean retention time (MRT), and other parameters of the test data were expressed as mean ± SD. Statistical analyses were performed using GraphPad Prism 5 (GraphPad Software, San Diego, CA, United States), and all experiments were repeated at least three times. The significance of the inter-group differences was determined by one-way ANOVA followed by the Kruskal-Wallis test in groups which did not fit a normal distribution. The experimental results are presented as mean ± SD or mean ± SEM, and significant differences are defined as *p* ≤ .05.

## Results

### Confirmation of ZLDI-8 derivative, NY-2, for follow-up studies and evaluation of hepatotoxicity

**FIGURE 2:**
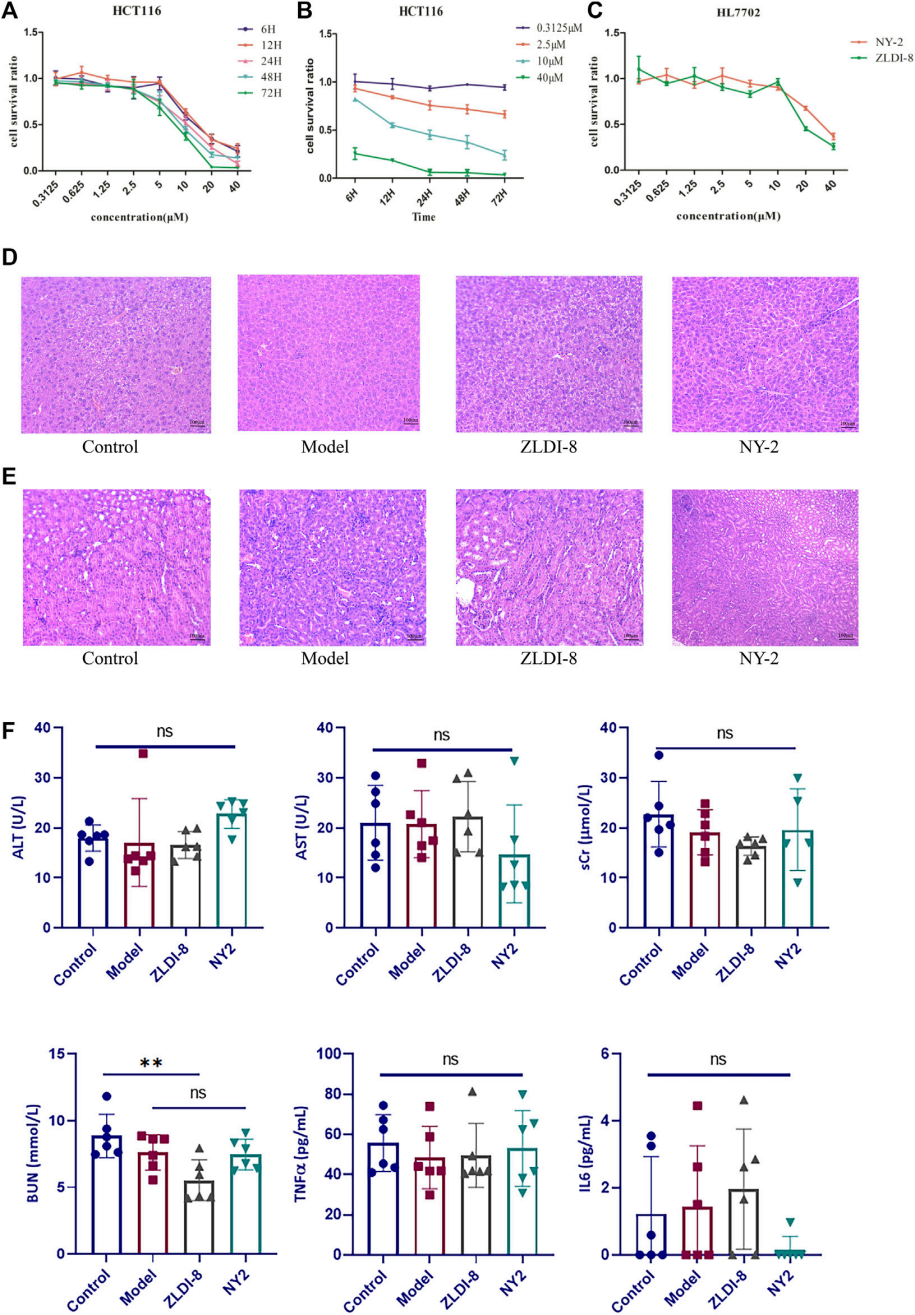
NY-2 shows preliminary antitumor activity, with no hepatotoxicity or nephrotoxicity. Relationship between concentration **(A)** and time **(B)** of NY-2 inhibition of HCT116 proliferation. Comparison of cytotoxic effects (48 h) of ZLDI-8 and NY-2 on normal liver tissue cells **(C)**. Schematic diagram of H&E staining (optical microscope, ×200) of mouse liver sections **(D)** and kidney sections **(E)**. Changes in serum AST, ALT, sCr, BUN, TNF-α, and IL-6 levels after ZLDI-8 and NY-2 administration (*n* = 5–6) **(F).** Mean ± SD, *significantly different.

The preliminary antitumor activity of the series of ZLDI-8 derivatives, NY-1 to -15 was screened by the MTT cell viability assay. The cytotoxicity outcomes are shown in Table 1. We found that the compound, NY-2, had the strongest anti-proliferative activity against HCT116 colorectal cancer cells, and therefore was selected for the experiments in this study (Figure 2A, B). The antitumor activity of NY-2 on HCT116 cells was concentration- and time-dependent, and slightly better than that of the original compound, ZLDI-8. At lower concentrations (≤10 μM), neither ZLDI-8 nor NY-2 had any obvious cytotoxic effects on normal cells, but at concentrations >10 μM, NY-2 showed slightly weaker toxic effects than ZLDI-8 (Figure 2C), which preliminarily proved that NY-2 was the safer of the two.

**TABLE 1:** IC_50_ values of ZLDI-8 and its derivatives, NY-1 to -15, in four cancer cell lines.

Compound	IC_50_(μM)			
	HCT116	LoVo	T47D	HepG2
NY-1	-	-	-	26.22
NY-2	5.35	5.82	34.71	20.03
NY-3	43.71	21.45	-	-
NY-4	27.53	41.83	25.41	-
NY-5	11.40	23.52	33.98	37.20
NY-6	38.76	28.74	53.74	40.75
NY-7	12.46	20.59	21.79	77.39
NY-8	-	38.06	10.30	-
NY-9	35.30	-	-	85.21
NY-10	26.03	86.43	23.31	66.67
NY-11	-	23.15	16.58	90.12
NY-12	20.03	-	40.36	-
NY-13	12.79	43.16	5.15	33.14
NY-14	-	-	74.33	-
NY-15	11.31	124.20	21.43	13.46
ZLDI-8	7.72	9.65	29.12	22.90

-, poor activity, cannot be unified; IC_50_, median inhibitory concentration

Compared with the control, the liver and kidney tissues from ZLDI-8- and NY-2-treated mice had no obvious pathological changes indicative of hepatotoxicity (Figure 2D) or nephrotoxicity (Figure 2E) after 7 days of injections. The assays of liver and kidney function showed that NY-2 treatment resulted in no significant differences in the levels of AST, ALT, sCr, and BUN compared with the control group. The only significant difference was found in the possible decrease of BUN with ZLDI-8, which proved that the lead compound, NY-2, may have less of an effect on renal function. In terms of the effect on immune inflammatory factors, we found that ZLDI-8 and NY-2 had no significant effect on IL-6 and TNF-α levels compared with the control group, and no significant effect on inflammation (Figure 2F). Moreover, we found that neither ZLDI-8 nor NY-2 had any overt effect on the overall antioxidant capacity (Supplementary Figure S2). As measured in rat liver microsomes and human liver microsomes, the residual NY-2 level was greater than that of ZLDI-8, indicating a greater metabolic stability (Supplementary Figure S3). An acute toxicity assay was assessed in KM mice by tail vein injection of NY-2 dissolved in saline at a dose of 1000 mg/kg (6 animals per group). The mice were kept under observation for 10 days, and no deaths occurred. Changes in body weight of mice are shown in Supplementary Figure S4, which further demonstrated NY-2’s strong safety profile in vivo. In summary, NY-2 showed no hepatotoxic or nephrotoxic tissue changes in mice, no effects on inflammation or total antioxidant capacity, and an excellent safety profile.

### Pharmacokinetic studies of ZLDI-8 and NY-2 in rats

By weighted 1/X^2^, the equation of the ZLDI-8 standard samples was y = 0.00127x ˗ 0.00155 for the concentration range of 20–1500 ng/mL of ZLDI-8, with correlation coefficient *r* ^2^ = 0.9979. The equation for the NY-2 standard samples was y = 0.00311x + 0.04139 (*r* ^2^ = 0.9987) for the concentration range of 100–2000 ng/mL of NY-2. The specificity was good with no interference from endogenous substances. The LLOQ of ZLDI-8 and NY-2 were 10 and 100 ng/mL, respectively, showing that the compounds have high sensitivity. The relative standard deviation (RSD) of the intra-day precision of the plasma samples of ZLDI-8 and NY-2 ranged from 1.90 to 14.90% and 1.75 to 2.77%, respectively; the RSD of the inter-day precision ranged from 5.7 to 13.6% and 4.43 to 10.43%; and the relative error (RE) of accuracy was ˗5.4 to ˗11.2% and ˗10.26 to ˗13.08%. The outcomes showed that both compounds had good inter-day and intra-day precision and accuracy. The recovery of ZLDI-8 was investigated at three concentrations: low (50 ng/mL), medium (500 ng/mL), and high (1000 ng/mL). The recovery of ZLDI-8 was more than 50% and the RSD was <15%. The percent extraction recovery of NY-2 at low (250 ng/mL), medium (1000 ng/mL), and high (1600 ng/mL) concentrations was >50%, higher than that of the parent compound ZLDI-8 where the RSD value was <15%. The two compounds met the requirements of biological sample analysis. The matrix effect test showed that the ratios of ZLDI-8 and NY-2 to the two peak areas of the internal standard were 85.1%–112.7% and 85.9%–105.8%, respectively, and the RSDs were <15%, which proved that there was no significant matrix effect. In addition, in terms of stability, the RE values of ZLDI-8 and NY-2 were in the range of ˗10.2% to ˗11.4% and ˗10.2% to ˗11.4%, respectively, with both RSD values <15%. The above outcomes demonstrated that the plasma samples containing the compounds were stable at room temperature for 12 h, in the auto-sampler at 4°C for 3 days, stored at ˗20°C for 14 days, and after three freeze-thaw cycles (˗20°C). Thus, both ZLDI-8 and NY-2 were stable under a variety of analytical conditions. Lastly, the 6000 ng/mL NY-2 plasma sample was diluted six times and measured. The RSD value was 6.3%, and the RE was ˗7.4 to ˗9.5, both within ±15%, which proved that NY-2 met the requirements of dilution stability (Table S1).

### Pharmacokinetic parameters of ZLDI-8 and NY-2 in rats

**FIGURE 3:**
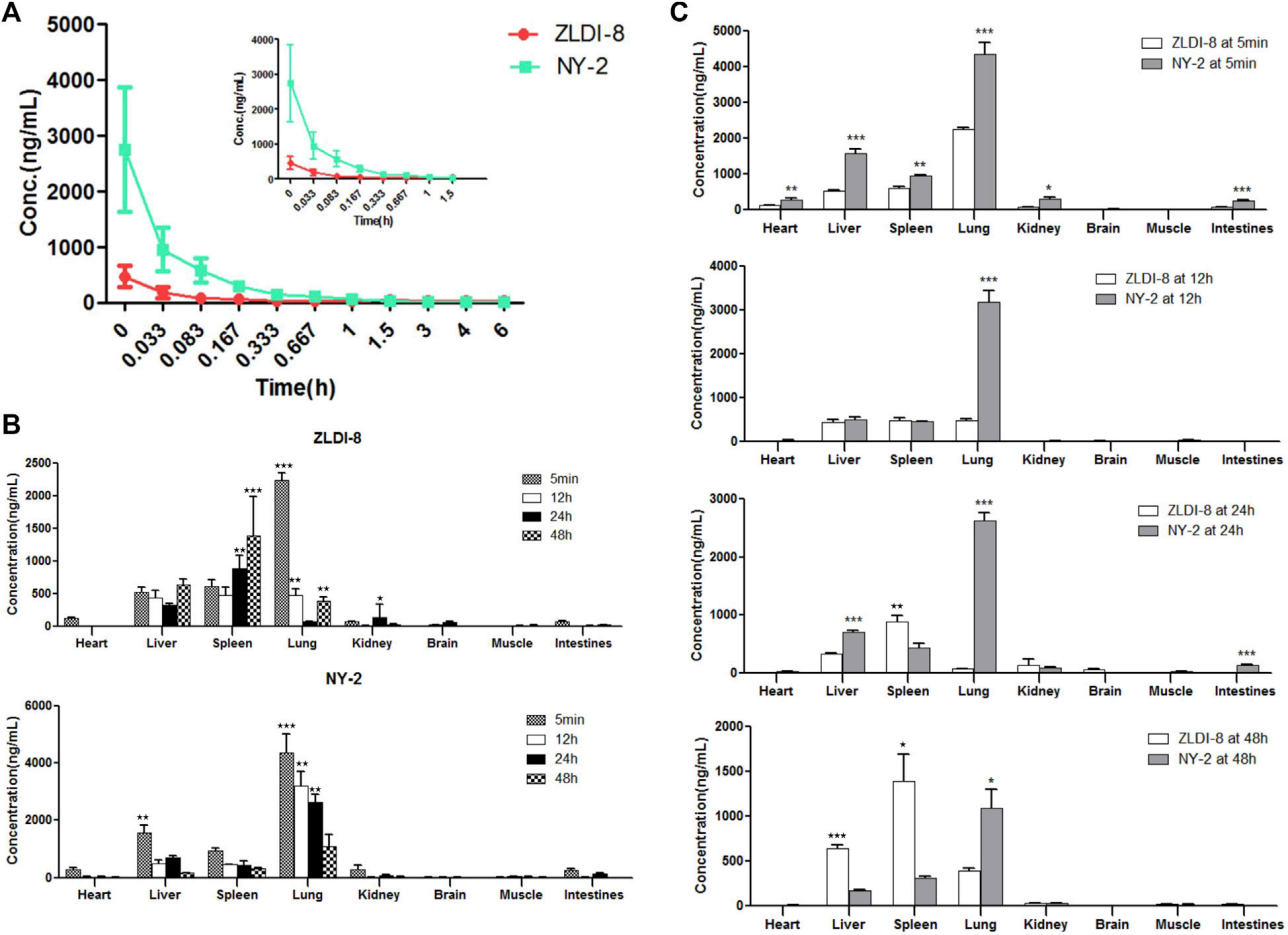
Pharmacokinetics and tissue distribution of ZLDI-8 and NY-2 in rats. Comparison of ZLDI-8 and NY-2 drug time curves **(A)**. Distribution of ZLDI-8 and NY-2 at 5 min, 12, 24, and 48 h **(B)**. Comparison of tissue distribution between ZLDI-8 and NY-2 at 5 min, 12, 24, and 48 h **(C)**. *p < .05, **p < .01, ***p < .001 (*n* = 6), *significantly different.

Plasma samples obtained after the SD rat tail vein injection of ZLDI-8 and NY-2 were processed and analyzed by HPLC-MS/MS, and the measured peak areas of ZLDI-8 and NY-2 were compared with the internal standard peak areas. The accompanying standard curves were used to calculate the plasma drug concentrations of each rat at different time points from the measured values. The plasma concentration-time data of ZLDI-8 and NY-2 at different times were fitted by DAS2.0 pharmacokinetic software, and the pharmacokinetic outcomes of the two compounds were obtained according to the two-compartment model and the pharmacokinetic parameters are shown in Table 2. The average plasma concentration-time curve after intravenous injection of 6 mg/kg compound ZLDI-8 and NY-2 was obtained by plotting the plasma concentrations against time. The average drug-time curve is shown in Figure 3A. Under the same dosage of the two compounds, the peak blood concentration (C_max_) of ZLDI-8 and NY-2 was 336.6 and 2750.7 ng/mL, respectively; the NY-2 group had 8.2 times the level of the ZLDI-8 group. The areas under the drug-time curves (AUC0-∞) of ZLDI-8 and NY-2 were 85.2 ng/mL × h ± 26.3 and 402.5 ng/mL × h ± 130.5; and the AUC0-t were 58.8 ng/mL × h ± 4.6 and 370.3 ng/mL × h ± 111.2, respectively; thus, NY-2 was 4.7 times and 6.3 times greater than ZLDI-8. The half-life periods (t_1/2_) of NY-2 and ZLDI-8 were 2.15 h and 0.59 h, respectively, and NY-2’s half-life was approximately 3.6-fold longer than ZLDI-8’s. The mean retention time (MRT_0-t_) was 1.07 h and 0.31 h, respectively, with NY-2 having an MRT_0-t_ 3.5 times lower than that of ZLDI-8. The plasma clearance (CL) of NY-2 was only 22.2% of ZLDI-8’s. In conclusion, the pharmacokinetics results after tail vein administration in rats showed that compared with ZLDI-8, the C_max_, AUC0-∞, AUC0-t, and t_1/2_ of NY-2 were significantly increased, while the MRT_0-t_ and CL were significantly decreased in the NY-2 group. Thus, we can conclude that the pharmacokinetic properties of NY-2 were significantly improved compared with those of ZLDI-8.

**TABLE 2:** Main pharmacokinetics parameters of compounds, ZLDI-8 and NY-2 in vivo.

Parameters	ZLDI-8	NY-2
C_max_ (ng/mL)	336.58 ± 19.39	2750.67 ± 1113.24^a^
AUC_0-t_ (ng/mL^a^h)	58.77 ± 4.62	370.25 ± 111.24^a^
AUC_0-∞_(ng/mL^a^h)	85.20 ± 26.28	402.45 ± 130.46^a^
t_1/2_(h)	0.59 ± 0.26	2.15 ± 0.62^a^
CL_Z_ (mL/h/kg)	126.60 ± 36.41^a^	28.13 ± 12.99
MRT_0-t_(h)	0.31 ± 0.02	1.07 ± 0.16^a^
V_Z_ (mL/kg)	98.88 ± 33.99	82.16 ± 28.13

Mean ± SD, *n* = 6.

^a^ Significant differences.

### Tissue distribution of ZLDI-8 and NY-2 in rats

The tissue distribution results of ZLDI-8 and NY-2 in SD rats at four different time points after administration by tail vein injection are shown in Figures 3B, C. Because of the rapid distribution, at 5 min after administration, the concentrations of ZLDI-8 and NY-2 in each tissue were in the following order: lung > liver > spleen > heart > kidney > intestines > brain > muscle, and at the initial stage of administration, the concentration of NY-2 in the heart, liver, spleen, lung, kidney, and small intestine was significantly higher than that of ZLDI-8. However, the concentration of both compounds in the brain was almost zero. The concentrations of both compounds decreased in each tissue over time. Comparing the concentrations of the two compounds in various tissues (Figure 3C) it can be seen that the concentration of NY-2 in the lung was higher than ZLDI-8 from the beginning of administration to 48 h. The concentration of NY-2 in the spleen was lower than ZLDI-8 at the beginning of 12 h, and the concentration of NY-2 in the liver and spleen was lower than ZLDI-8 at 48 h, indicating that the concentration of compound NY-2 in the tissues decreased slowly at first and then faster than ZLDI-8 after administration, and the safety of NY-2 was thus greater than with ZLDI-8. To summarize, the tissue distribution results show that the concentration of NY-2 after tail vein administration is the highest in the lung, meaning that NY-2 has the highest affinity to the lung, which suggests that it can be useful as a therapeutic agent to target lung cancer. On this basis, follow-up studies will evaluate the activity of NY-2 against NSCLC in vitro and in vivo.

### Cytotoxic effects of NY-2 and ZLDI-8 on NSCLC cells

**FIGURE 4:**
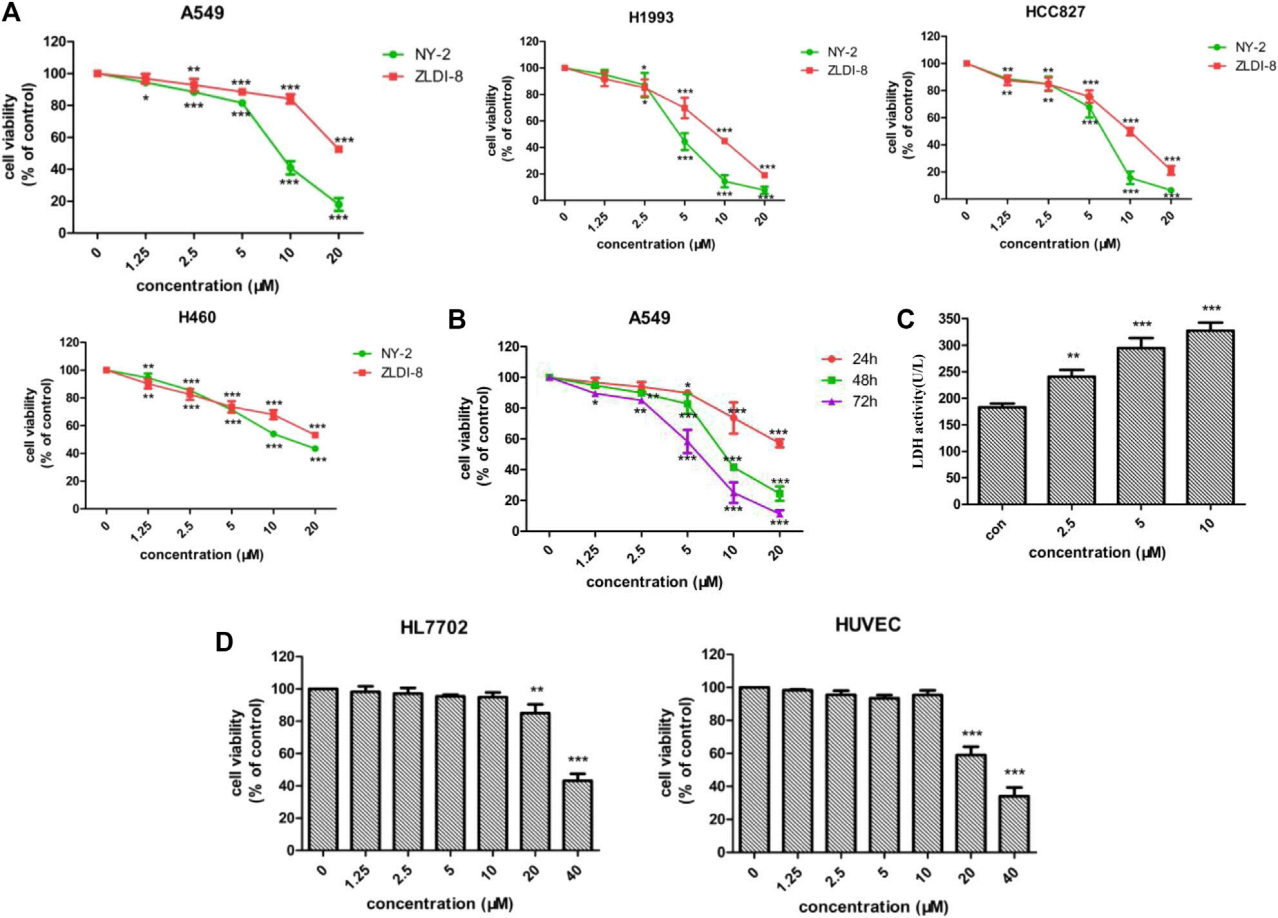
Cytotoxic effects of NY-2 and ZLDI-8 on NSCLC cells. Effect of NY-2 and ZLDI-8 on the survival of A549, H1993, HCC827, and H460 (NSCLC) cells at 48 h **(A)**. NY-2 effect on survival of A549 cells at 24, 48, and 72 h **(B)**. NY-2 effect on LDH activity of A549 cells **(C)**. NY-2 effect on survival of HL7702 and HUVECs (normal human cells) for 48 h **(D)**. *p < .05, **p < .01, ***p < .001 (*n* = 3, mean ± SD).

First, the effects of NY-2 and ZLDI-8 on the survival rates of four NSCLC cell lines, A549, H1993, HCC827, H460, were compared by the MTT cytotoxicity assay. The various types of NSCLC cells were incubated with increasing concentrations of NY-2 and ZLDI-8 (1.25, 2.5, 5, 10, and 20 μM), and the cell viability at 48 h was measured. As shown in Figure 4A, compared with the control group, NY-2 and ZLDI-8 could inhibit the survival of A549, H1993, HCC827, and H460 cells in a concentration-dependent manner. NY-2 was significantly more cytotoxic against all four kinds of NSCLC cells, and its IC_50_ values are summarized in Table 3. The above results indicate that the derivative compound, NY-2, not only has improved pharmacokinetic properties compared with ZLDI-8, but also is better at inhibiting the survival of NSCLC cells in vitro. As shown in Figure 4B, NY-2 inhibited the survival of A549 cells in a time- and concentration-dependent manner. Considering that the culture time might be too long, NY-2 was selected for 48 h treatment at increasing concentrations of 2.5, 5.0, and 10 μM as the experimental conditions for subsequent in vitro experiments. As shown in Figure 4C, when A549 cells were treated with increasing concentrations of NY-2 for 48 h, the LDH activity in cell culture supernatants increased in a concentration-dependent manner compared with the control group, which proved that the degree of cell damage was also affected by NY-2 concentration. To determine if NY-2 had a cytotoxic effect on normal human cells, normal human hepatocyte line (HL-7702) and HUVECs were incubated for 48 h with NY-2 at 1.25, 2.5, 5, 10, 20, and 40 μM, and cell viability was measured. As shown in Figure 4D, NY-2 had no obvious cytotoxic effects on HL-7702 or HUVEC cells at a concentration of 10 μM or below, while the viability of normal human lung cells incubated with ≥20 μM NY-2 was comparable to that of the control group with a significant difference (*p* < .01). Therefore, the maximum concentration in subsequent in vitro experiments was set at <10 μM, which showed no obvious cytotoxicity to normal cell lines.

**TABLE 3:** IC50 values of NY-2 and ZLDI-8 on four kinds of NSCLC cells (48 h).

Cell lines	IC50(μM)	
	NY-2	ZLDI-8
A549	8.95 ± .58^a^	23.06 ± 2.27
H1993	4.78 ± .64^a^	8.33 ± 1.01
HCC827	5.98 ± .75^a^	9.39 ± .99
H460	13.53 ± .58^a^	25.22 ± 2.00

^a^ Significant differences.

### In vitro activity of NY-2 against NSCLC and signaling pathway identification

**FIGURE 5:**
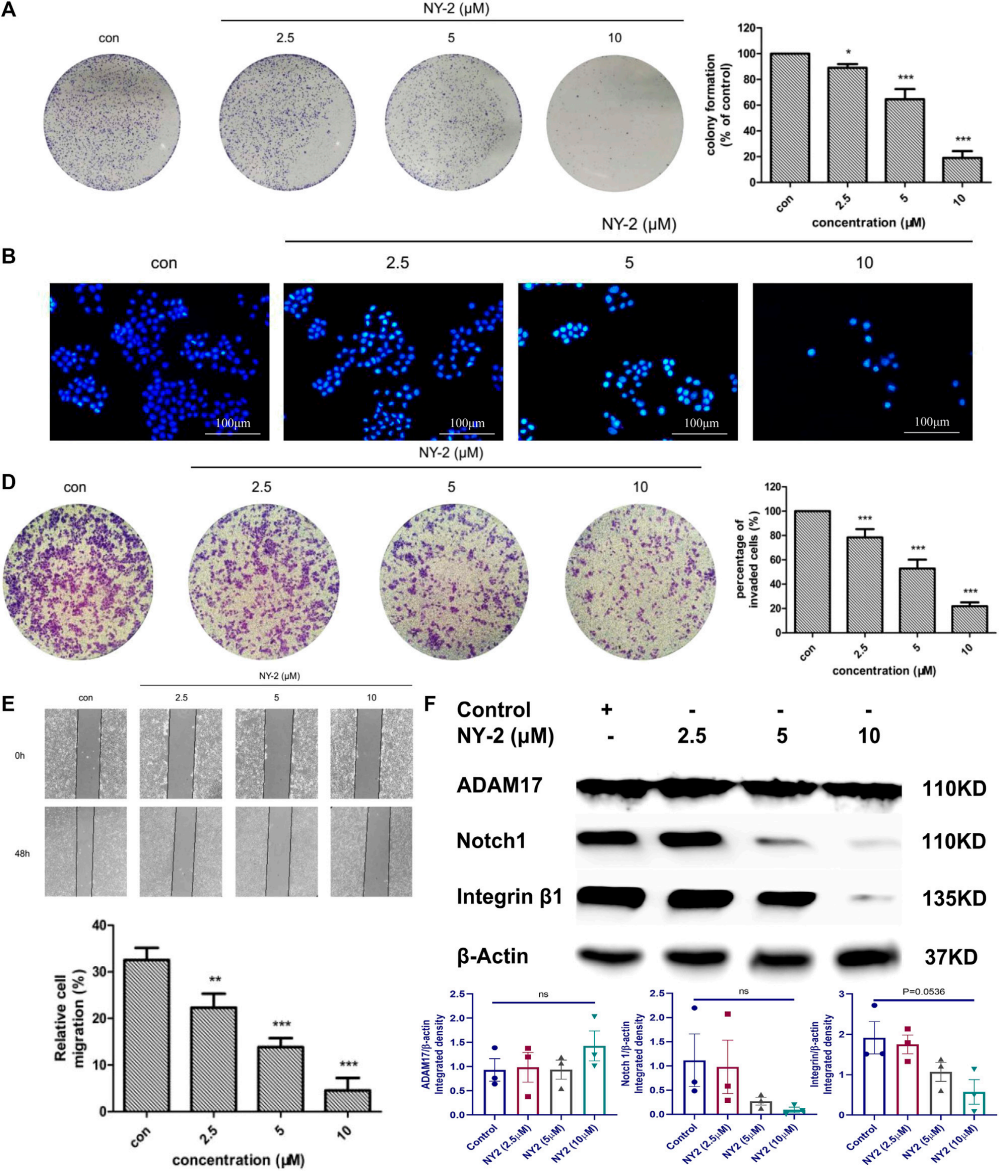
In vitro activity of NY-2 against NSCLC. Effect of NY-2 on colony formation of A549 cells **(A)**. NY-2 promotes apoptosis of A549 cells (Hoechst staining) **(B)**. NY-2 promotes apoptosis of A549 cells (annexin-V/PI double staining) **(C)**. NY-2 inhibits invasion of A549 cells **(D)**. NY-2 inhibits migration of A549 cells **(E)**. Western blot showing NY-2 reduces expression of Notch1 and integrinβ1 protein in dose-dependent manner **(F)**. *p < .05, **p < .01, ***p < .001 (*n* = 3, mean ± SD).

We found that NY-2 could inhibit colony formation of A549 cells in a concentration-dependent manner after 9 days of growth (Figure 5A). The Hoechst 33342 nuclear staining revealed that NY-2 triggered apoptosis in A549 cells, compared with the untreated control group in which the nuclei were normal with uniform low-intensity blue fluorescence. As the concentration of NY-2 was increased, the number of A549 cells gradually decreased, and the nuclei presented morphological changes such as pyknosis and hyperchromatic staining, which showed a concentration-dependent change (Figure 5B). To further evaluate the apoptosis-promoting effect of NY-2 on A549 cells, the annexin-V/PI double staining method was used to detect apoptotic cells. As shown in Figure 5C, compared with the control group, the percentage of A549 cells in apoptosis was significantly increased from 0.86 ± .05% to 5.45 ± 3.28% at 5 μM and 20.29 ± 3.11% at 10 μM after incubation with NY-2 for 48 h (*p* < .05). In terms of the effect of NY-2 on the invasion and migration of A549 cells, the number of A549 cells that penetrated the basement membrane of the transwell chamber was significantly less than that of the control group (Figure 5D), and NY-2 reduced the migration of A549 cells in the wound-healing assay relative to control. Therefore, NY-2 could inhibit the invasion and migration of A549 cells in a concentration-dependent manner (Figure 5E). The above outcomes demonstrated that NY-2 could inhibit the proliferation, invasion, and migration of A549 cells and promote their apoptosis in vitro. To investigate the effects of NY-2 on signal transduction, the Notch1 and integrinβ1 signaling pathways were examined. As shown in Figure 5F, NY-2 reduced the expression of Notch1 and integrinβ1in a dose-dependence manner, most likely by inhibiting the activation of ADAM17 rather than its expression. Thus, NY-2 may block NSCLC proliferation by inhibiting ADAM17 activation and the Notch1 and integrinβ1 signaling pathways.

### In vivo activity of NY-2 against NSCLC

**FIGURE 6:**
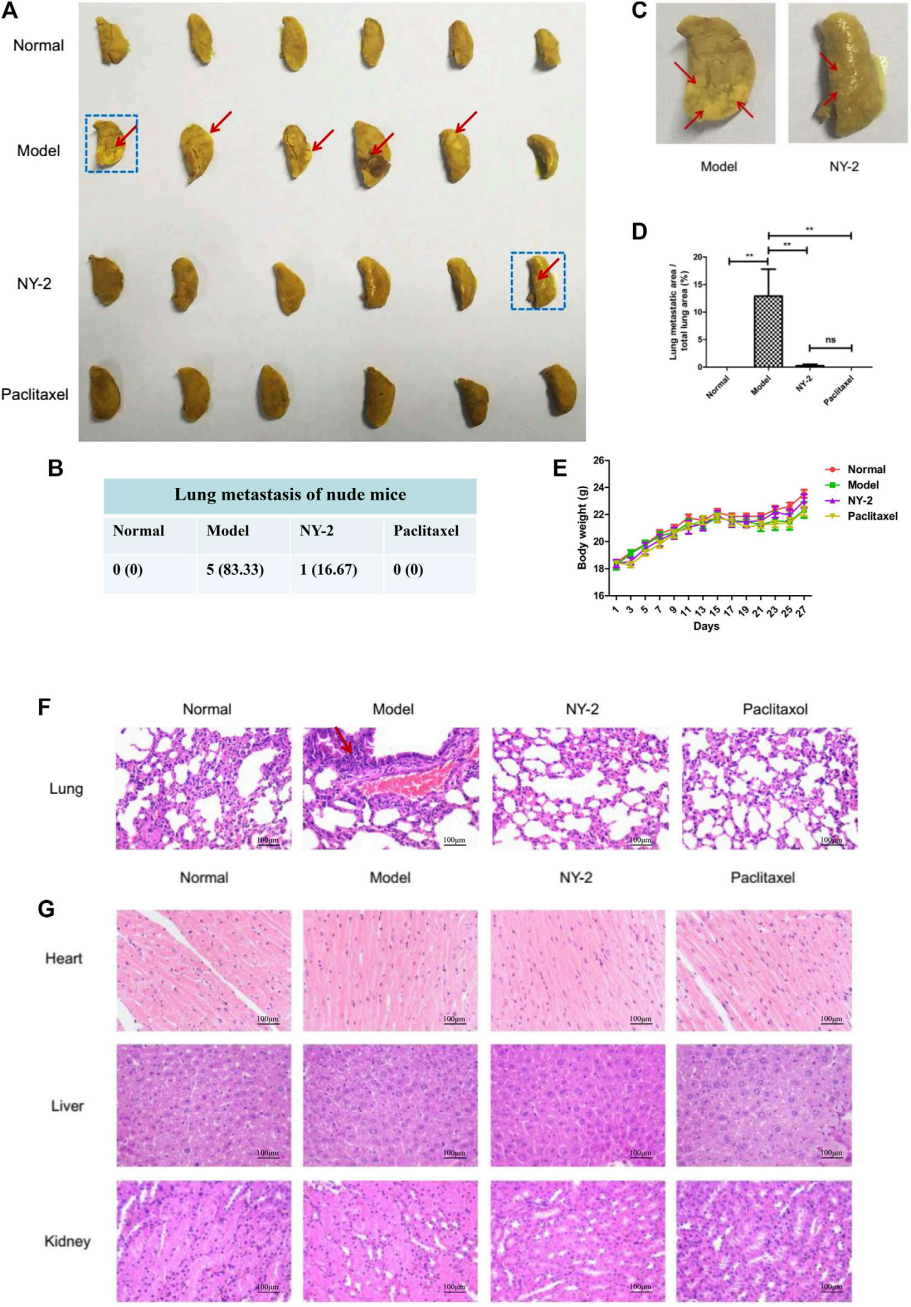
In vivo activity of NY-2 against NSCLC. The left lungs of nude mice from each group after staining with Bouin’s solution **(A)**. Lung metastases in nude mice from each group **(B)**. Enlarged left lung marked with blue box from Figure **(A) (C)**. Percentage of the metastatic tumor area relative to the total lung area of nude mice in each group **(D)**. Effect of NY-2 on body weight of tumor-bearing nude mice **(E)**. H&E staining of lung tissue sections **(F)**. H&E staining of heart, liver, and kidney tissue sections **(G)**. *p < .05, **p < .01, ***p < .001 (*n* = 6, mean ± SEM).

The in vivo antitumor activity of NY-2 needs further validation in animal models. There were no obvious abnormalities in the diet, activity levels, or feces of the nude mice in each group, and no mice died during the experimental period. After 28 days, the nude mice were euthanized, and the main organs were removed. Figure 6A shows the left lungs from the nude mice in each group. It can be seen that small nodular metastatic tumors had formed on the lung surface of five mice in the model group, with a tumorigenicity rate of 83.3%. The shapes and sizes of each metastatic tumor were very different, and they were yellowish convex after being stained with Bouin’s solution (Figure 6C). The fourth lung from left to right in the model group was necrotic. No metastatic tumors were found in the paclitaxel group and only one mouse in the NY-2 group had metastatic tumors on the lung surface (Figure 6B). The presence of growing tumors and the injected medication had no significant effect on the weight of mice in each group (Figure 6E). The statistical results of the areas of lung surface metastases are shown in Figure 6D. The average percentage of the metastatic lung surface area to the total lung area in the model group was 12.91 ± 11.93%, and the average percentage of metastatic lung surface area to the total lung area in the NY-2 group was 0.25 ± .62)%, and the difference between them was statistically significant (*p* < .01). Compared with the blank control group, the viscera-to-body ratios of the lungs and livers in the model group were increased (*p* < .05), but the viscera-to-body ratios of the heart and kidneys were not statistically different (*p* > .05). Compared with the model group, the liver-to-body ratio of the NY-2 group and the paclitaxel group was decreased, and the difference was statistically significant (*p* < .05). Compared with the normal control group, there was no statistical difference (*p* > .05, Supplementary Table S2). The results of H&E staining of the lungs are shown in Figure 6F. The lung tissue in the blank control group had a reticular structure, while the model group showed high-density tumor cell clusters with disorderly arrangement, larger nuclei, and darker staining. After administration of NY-2 and paclitaxel, there was no infiltration of A549 cells in the lung tissue, which shows that NY-2 can inhibit the metastasis of A549 cells. The H&E staining of other organs showed no abnormal histomorphological changes in the heart, liver, and kidney of nude mice in each group. Tail vein injection at a dose of 6 mg/kg, NY-2 had no obvious toxic effects on the heart, liver or kidney (Figure 6G). Thus, NY-2 demonstrates clear anti-NSCLC activity in nude mice with no obvious organ toxicity.

## Discussion

In this study, we found by primary screening that among 15 derivatives of ZLDI-8, NY-2 at concentrations below 10 μM had the best antitumor activity. In vivo experiments revealed that the pharmacokinetics parameters of NY-2 were better than the parent compound, ZLDI-8. Tissue distribution studies showed that the concentration of NY-2 was highest in the lung, which led to the hypothesis that NY-2 could be effective in the treatment of lung cancer. We also found that NY-2 had a greater inhibitory effect on NSCLC cell lines than ZLDI-8, by preventing colony formation, invasion, and migration, and increasing LDH activity and apoptosis in a concentration-dependent manner, which act on Notch1 and Intergrinβ1 pathways. Lastly, we showed that tail vein injection of NY-2 could inhibit the formation of lung metastases without significant toxicity to other major organs. In conclusion, the effectiveness and safety of the ZLDI-8 derivative, NY-2, for NSCLC are very good, and NY-2 has great potential as an effective NSCLC treatment compound targeting the Notch signaling pathway.

We found that the pharmacokinetic properties of NY-2 were significantly improved compared to ZLDI-8 with distinct advantages. These effects may be related to the strong electron-withdrawing property of the fluorine atom added to the derivative, which reduced the oxidative metabolism of the enzymes in the metabolic pathway in vivo (Kuhn et al., 2006; Antonov et al., 2016), so that the CL_Z_ rate of NY-2 in rats was significantly lower (only 1/5) than that of ZLDI-8. In addition, the t_1/2_ and MRT_0-t_ of NY-2 were higher than with ZLDI-8. These results indicate that NY-2 can maintain a relatively high plasma concentration for a longer time than ZLDI-8, which helps the compound accumulate in target tissues and exert a stronger targeted anticancer effect. From the pharmacokinetics results, it can be seen that the compounds will quickly enter the tissue and stay in the tissue after entering the blood, while the retention time in the blood is very short.

The tissue distribution experiment, revealed that ZLDI-8 and NY-2 were highly concentrated in the lungs, which suggested that this high affinity for the lung can allow ZLDI-8 and NY-2 to target lung tumors, and may also be related to the abundant blood flow of the lung itself. The second highest affinity is the liver, indicating that the high drug concentration of ZLDI-8 and NY-2 in liver tissue could be used for the treatment of hepatocellular carcinoma (Zhang et al., 2017; Zhang et al., 2018). It has been suggested that ZLDI-8 and NY-2 are mainly metabolized by the liver, but they do not appear to have significant hepatotoxicity. Moreover, our previous study found that ZLDI-8 also had a therapeutic effect against lung metastasis from hepatocellular carcinoma (Lu et al., 2020), which is consistent with our findings. The concentration of ZLDI-8 and NY-2 in rat brain tissue was very low, indicating that they do not readily penetrate the blood-brain barrier.

NY-2 has a 2-mercaptobarbituric acid structure, a substituted indole, and a substituted phenol, which may be responsible for the antitumor activity. Lee et al. (2020) found two derivatives of thiobarbituric acid with potential usefulness as therapeutic agents in lung cancer. Moreover, Jia et al. (2020) reported that indole derivatives were a significant source of novel anticancer agents against drug-resistant tumors. In summary, the antitumor activity of NY-2 has been well established and supported by other research. We found that NY-2 could significantly inhibit the survival of four different NSCLC cell lines compared with ZLDI-8. The NY-2 derivative not only showed improved pharmacokinetic properties compared with ZLDI-8, but also inhibited the viability of NSCLC cells in vitro. However, we only used one cell line for the follow-up experiments, which may limit the general applicability of the compound. In animal experiments, we found that five mice in the model group had small nodular metastases on the surface of the lungs, with a tumor formation rate of 83.3%, while one nude mice in the model group had no metastases on the surface of the lungs, which may be related to the shorter experimental period and a smaller number of cells transplanted. In the in vivo experiment, the total time from modeling to euthanasia was 29 days, and each nude mouse was injected with 0.2 mL of cell suspension (2×10^6^ cells/mouse). An excessive cell concentration could cause pulmonary embolism in mice, and a large injection volume could result in discomfort or even death due to the heavy heart load. The inoculation volume of 0.2 mL is relatively large, and the success rate of modeling could be improved by extending the experimental cycle in subsequent experiments.

There are some limitations in our research. First, the experiments only proved that NY-2 was effective and safe for the treatment of NSCLC, but its mechanism has not been discussed in depth, only western blot experiments were conducted to verify its action on Notch1 and Intergrinβ1 signaling pathways. Also, we only showed that NY-2 could have a pharmacological role in anti-NSCLC treatment. In future, we will test NY-2 on other types of tumors, such as colorectal and liver. Although NY-2 has excellent pharmacokinetics, we do not know if it has long-term toxicity, and further research is needed. For example, it may cause cardiac toxicity when it is used in the clinic. NY-2 could also be packaged in a targeting material to become a molecular targeting drug with better selectivity for tumor tissues.

## Conclusion

In conclusion, the pharmacokinetics parameters and tissue distribution of NY-2, the most effective derivative of ZLDI-8, were determined and compared to its parent compound: its pharmacokinetics properties were improved. Both NY-2 and ZLDI-8 were highly distributed in the lung, and almost lacking in the brain, so we conclude that NY-2 targets the lung. The in vitro and in vivo results with NY-2 against NSCLC have shown for the first time that NY-2 can inhibit the occurrence and development of NSCLC by suppressing the activation of Notch1 and Intergrinβ1 and blocking the EMT. Moreover, NY-2 showed no significant toxicity to major organs. Therefore, compared with parent compound ZLDI-8, the activity and safety of compound NY-2 have both been improved, and NY-2 can take its place as a potential antitumor agent for NSCLC treatment, targeting the Notch1 and integrinβ1 signaling pathways.

## Data Availability

The raw data supporting the conclusion of this article will be made available by the authors, without undue reservation.

## Supplementary material

SUPPLEMENTARY FIGURE S1 Determination of the purity of the compounds **(A)** ZLDI-8 and **(B)** NY-2.

SUPPLEMENTARY FIGURE S2 Total antioxidant capacity of ZLDI-8 and NY-2.

SUPPLEMENTARY FIGURE S3 Comparison of the metabolic stability of ZLDI-8 and NY-2 (100 μM) in rat liver microsomes (RLMs) and human liver microsomes (HLMs).

SUPPLEMENTARY FIGURE S4 In vivo acute toxicity experiment of compound NY-2; the weight changes of mice within ten days.

SUPPLEMENTARY FIGURE S5 Original western blots of compound NY-2.

SUPPLEMENTARY TABLE S1 Summary results of pharmacokinetic studies.
