# Supplementary figures and images for: Pharmacokinetics, tissue distribution, and antitumor activity of a novel compound, NY-2, in non-small cell lung cancer
Source: Front Pharmacol. 2023 Jan 16;13:1074576. doi: 10.3389/fphar.2022.1074576 (PMC9884808; doi:10.3389/fphar.2022.1074576)

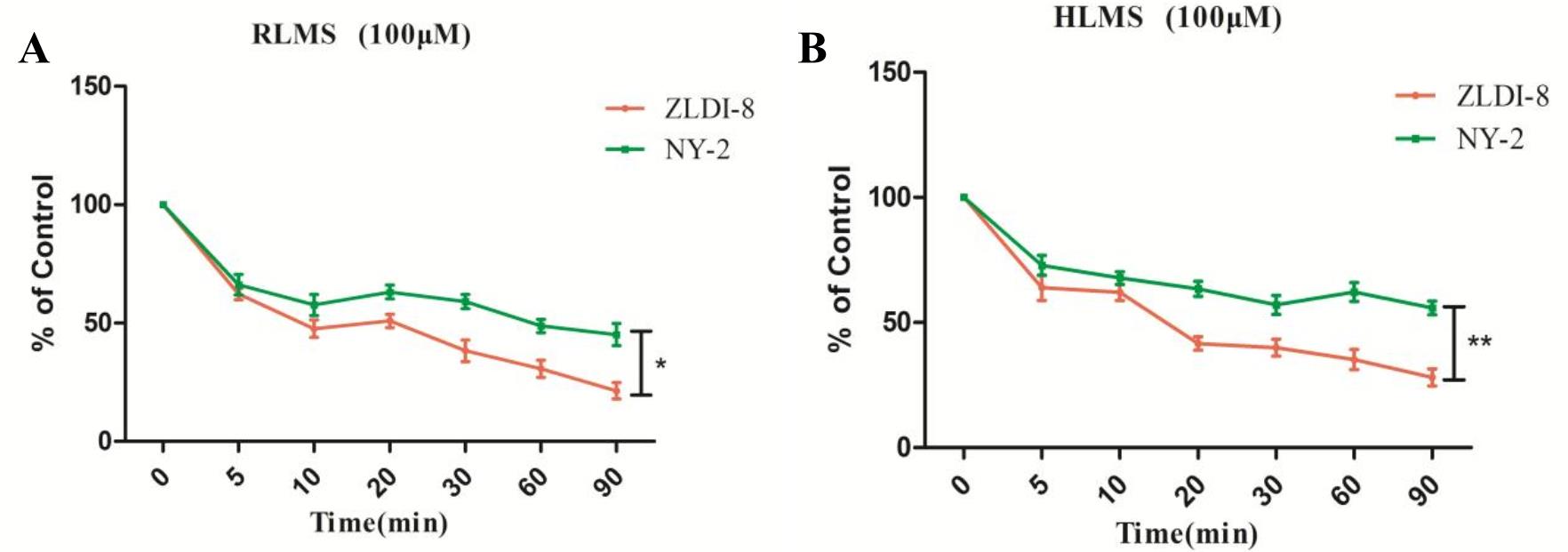

Supplement: Supplementary file 1 [file Image3.JPEG]

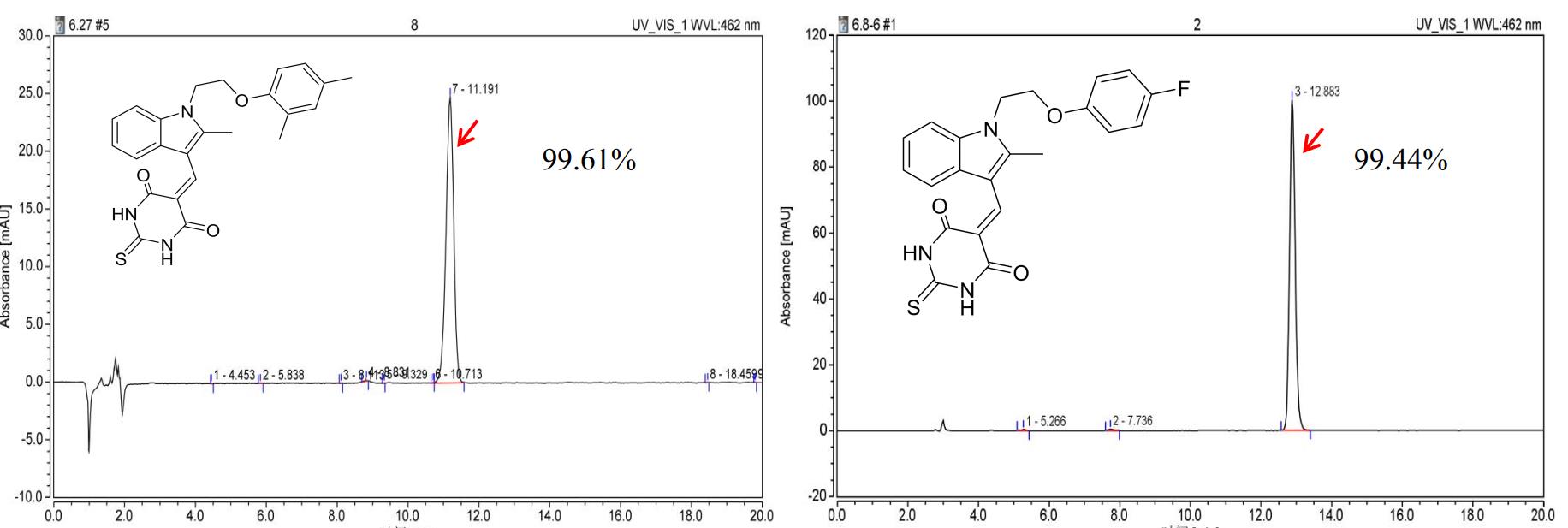

Supplement: Supplementary file 2 [file Image1.JPEG]

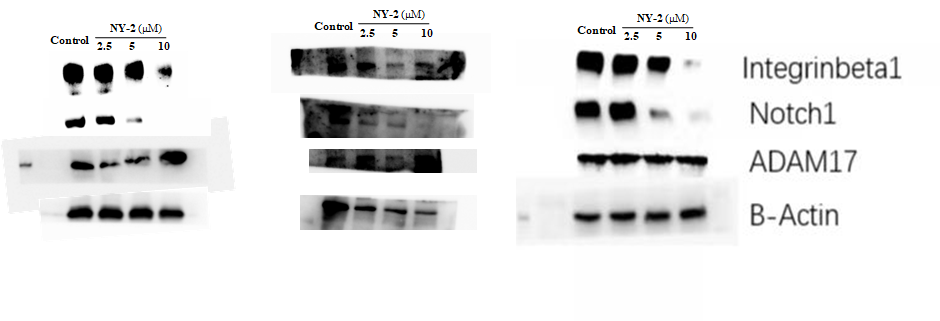

Supplement: Supplementary file 4 [file Image5.PNG]

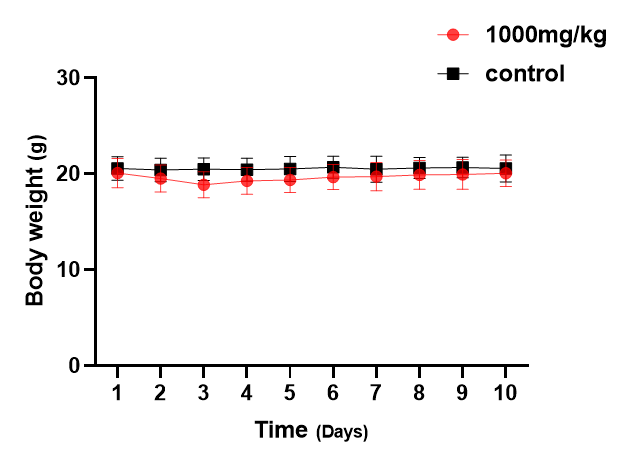

Supplement: Supplementary file 5 [file Image4.PNG]

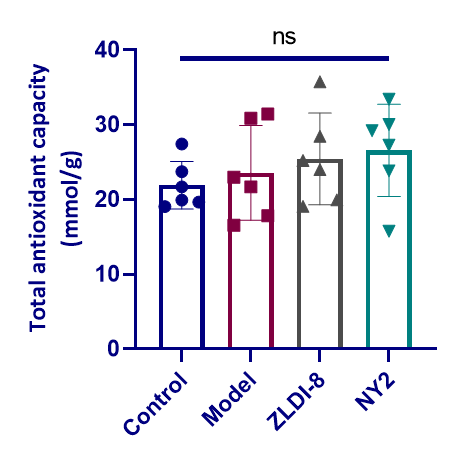

Supplement: Supplementary file 7 [file Image2.PNG]
