# Supplementary material for: Pharmacokinetics, tissue distribution, and antitumor activity of a novel compound, NY-2, in non-small cell lung cancer
Source: Front Pharmacol. 2023 Jan 16;13:1074576. doi: 10.3389/fphar.2022.1074576 (PMC9884808; doi:10.3389/fphar.2022.1074576)
Supplement: Supplementary file 6 [file Table1.DOC]

**Table S1 The visceral coefficient of nude mice in each group**

|  | **Normal** | **Model** | **NY-2** | **Paclitaxel** |
| --- | --- | --- | --- | --- |
| Body weight before dissection(g) | 23.79 ± 0.28 | 22.70 ± 0.53 | 23.48 ± 0.50 | 22.72 ± 0.32 |
| Quality of the lung(g) | 0.14 ± 0.00 | 0.17 ± 0.02 | 0.16 ± 0.01 | 0.15 ± 0.01 |
| Lung as a proportion of body weight(10^-2^) | 0.57 ± 0.01 | 0.77 ± 0.09* | 0.67 ± 0.03 | 0.64 ± 0.02 |
| Quality of the heart(g) | 0.13 ± 0.00 | 0.13 ± 0.00 | 0.13 ± 0.01 | 0.14 ± 0.01 |
| Heart as a proportion of body weight(10^-2^) | 0.56 ± 0.01 | 0.55 ± 0.02 | 0.56 ± 0.03 | 0.58 ± 0.0.03 |
| Quality of the liver(g) | 1.31 ± 0.03 | 1.35 ± 0.04 | 1.28 ± 0.04 | 1.29 ± 0.06 |
| Liver as a proportion of body weight(10^-2^) | 5.50 ± 0.10 | 5.93 ± 0.18* | 5.45 ± 0.05# | 5.45 ± 0.20# |
| Quality of the kidney(g) | 0.42 ± 0.01 | 0.41 ± 0.01 | 0.41 ± 0.01 | 0.41 ± 0.01 |
| Kidney as a proportion of body weight(10^-2^) | 1.77 ± 0.02 | 1.81 ± 0.02 | 1.74 ± 0.03 | 1.74 ± 0.03 |

Compared with normal group, *p < 0.05, **p < 0.01, ***p < 0.001; Compared with model group, #p < 0.05, ##p < 0.01, ###p < 0.001（n=6, mean ± SEM）
