# Supplementary material for: Pharmacokinetics, tissue distribution, and antitumor activity of a novel compound, NY-2, in non-small cell lung cancer
Source: Front Pharmacol. 2023 Jan 16;13:1074576. doi: 10.3389/fphar.2022.1074576 (PMC9884808; doi:10.3389/fphar.2022.1074576)
Supplement: Supplementary file 8 [file Table2.doc]

**Comparison of metabolic stability between ZLDI-8 and NY-2 by in vitro liver microsome incubation**

Rat liver microsomes (RLMS) and human liver microsomes (HLMs) were purchased from RILD and BioIVT, respectively.The metabolic stability of 100 μm Zldi-8 and NY-2 in rat and human liver microsomes was investigated. Zldi-8/NY-2(100 ΜM)10 μl, mgcl210 ΜL, RLMs/HLMs 62.5 μl, PBS (ph 7.4)117.5 μl, NADPH 50 ΜL were added to the metabolic incubation system in ice bath, respectively.After adding in sequence, the incubation systems were pre-incubated for 3 min in a 37 ° C incubator, and then NADPH was added to start the reaction, and the incubation systems were removed at 0 min, 5 min, 10 min, 20 min, 30 min, 60 min, 90 min, 50 ΜL incubation solution was taken out respectively, and the reaction was stopped by rapidly adding equal volume of ice acetonitrile solution containing propranolol hydrochloride (internal standard) , vortex for 3 min, centrifugation at 4 ° C, 9500 rpm for 10 min, the content of the supernatant was determined by HPLC.The experiment was repeated three times in parallel.
